# Acquired Factor VIII (FVIII) Deficiency: A Case of Idiopathic Hemorrhage

**DOI:** 10.7759/cureus.93918

**Published:** 2025-10-06

**Authors:** Ahmad Khalil, Dina Foudeh, Ismael Nassar, Mokeem Nusir, Waleed Alameh

**Affiliations:** 1 Department of Oncology, Islamic Hospital, Amman, JOR; 2 Department of Internal Medicine, Islamic Hospital, Amman, JOR; 3 Department of Radiology, Islamic Hospital, Amman, JOR; 4 Faculty of Medicine, An-Najah National University, Nablus, PSE

**Keywords:** acquired factor viii deficiency, acquired hemophilia a (aha), factor viii inhibitor, prolonged activated partial thromboplastin time, spontaneous intramuscular hematoma

## Abstract

We report the case of a 34-year-old female with no prior medical history or known bleeding disorders who presented with a large, spontaneous right arm hematoma developing gradually over 10 days, without preceding trauma. She also had multiple ecchymoses and a history of a right calf hematoma three months earlier, initially misdiagnosed and managed as deep venous thrombosis and later as muscle cramp. Laboratory evaluation revealed anemia (hemoglobin: 9.9 g/dL) and a markedly prolonged activated partial thromboplastin time (aPTT) that failed to correct with mixing studies. Factor VIII activity was severely reduced at 4%, with an inhibitor titer of 3.1 Bethesda units, confirming acquired hemophilia A. Imaging demonstrated an intramuscular hematoma within the biceps. The patient was treated with high-dose intravenous methylprednisolone for three days, a single dose of cyclophosphamide, and transitioned to oral prednisolone and azathioprine. Within 24 hours, her aPTT improved to near-normal, and at one-month follow-up, she demonstrated complete resolution of symptoms, normalized FVIII activity, and no recurrence. This case underscores the importance of prompt recognition and combined laboratory, imaging, and clinical assessment in diagnosing acquired hemophilia, particularly in atypical presentations without mucocutaneous bleeding. Early targeted immunosuppressive therapy can result in rapid inhibitor eradication and favorable outcomes.

## Introduction

Acquired factor VIII (FVIII) deficiency, also known as acquired hemophilia A (AHA), is a rare entity in which antibodies target FVIII, causing severe and potentially fatal bleeding [1].

In nearly 50% of cases, the cause of AHA remains idiopathic. In the remaining cases, AHA is associated with postpartum status, autoimmune diseases, hematologic malignancies, infections, or medications.

The estimated incidence of AHA is one to four cases per million per year. Severe bleeding episodes occur in up to 90% of affected individuals, with reported mortality rates ranging from 8% to 22%. The incidence increases with age and is exceptionally rare in children. There are two incidence peaks for FVIII autoantibodies. The first, occurring between 20 and 30 years of age, is likely due to postpartum-related inhibitors, making AHA more common in women aged 20-40 years. The second, more prominent peak occurs between 68 and 80 years of age [2].

The clinical presentation of AHA differs from congenital hemophilia in that most patients with AHA present with bleeding in the skin, muscles, soft tissues, and mucous membranes. Common manifestations include epistaxis, gastrointestinal, urologic, and retroperitoneal hemorrhages, as well as postpartum bleeding. In contrast, hemarthrosis - characteristic of congenital FVIII deficiency - is uncommon in AHA [3,4].

Due to its distinct bleeding pattern, early recognition of AHA is crucial for timely intervention. Treatment primarily aims to control acute bleeding, eliminate inhibitors, restore FVIII levels, and administer immunosuppressive therapy [1,5].

## Case presentation

This is a 34-year-old female with no known medical conditions, no history of bleeding disorders, and not on anticoagulants or antiplatelets. Her last delivery was 10 months ago.

She presented with a large hematoma involving her entire right arm that developed gradually over 10 days and was not preceded by trauma. Additionally, she had multiple ecchymoses of varying ages on her arm. She denied any bleeding from other sites, including epistaxis, hemoptysis, melena, hemarthrosis, or hematemesis.

Laboratory investigations (Table 1) revealed a hemoglobin (Hgb) level of 9.9 g/dL and a prolonged activated partial thromboplastin time (aPTT) of 52.7 seconds, which did not correct with a mixing study (post-mixing aPTT after one hour was 45.1 seconds). FVIII activity was reduced to 4%, with an inhibitor titer of 3.1 Bethesda units (BU). Anti-nuclear antibody (ANA) and rheumatoid factor (RF) tests were negative.

**Table 1:** Laboratory results before, during, and after hospitalization aPTT: activated partial thromboplastin time; Hgb: hemoglobin

Labs	Timing	Result	Normal range
Hgb	Current presentation	9.9 g/dL	12-15 g/dL
aPTT		52 sec	26-40 sec
Factor VIII		4%	60-150%
ANA (anti-nuclear antibody)		Negative	
RF (rheumatoid factor)		Negative	
aPTT	1 Day after treatment	34 sec	26-40 sec
aPTT	One month follow-up	29.9 sec	26-40 sec
Factor VIII		69%	60-150%

**Figure 1:**
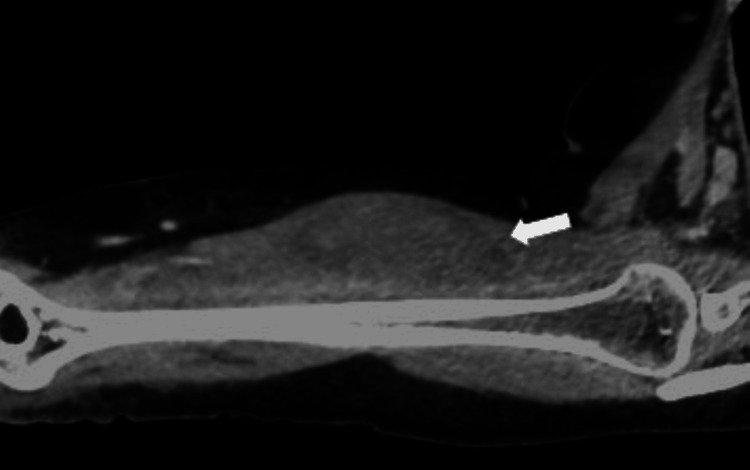
Selected sagittal oblique CT image of the biceps muscle The CT image in the venous phase demonstrates a well-defined hypoattenuating intramuscular collection (white arrow) within the biceps muscle.

The patient underwent a contrast-enhanced CT scan of the right arm, which revealed an intramuscular hematoma within the biceps muscle (see Figure 1).

She was treated with intravenous methylprednisolone at a dose of 1 g daily for three consecutive days, followed by a single dose of intravenous cyclophosphamide (1 g). One day after treatment, her aPTT improved to 34 seconds.

Following treatment with intravenous methylprednisolone and cyclophosphamide, she was put on oral prednisolone 60 mg daily for one month and then tapered gradually, and oral azathioprine 50 mg twice daily.

The patient had a complete resolution of the hematoma, and the inhibitor was eradicated. At the one-month follow-up, her FVIII level had normalized to 69%, and her aPTT was 29.9 seconds, with no recurrence of symptoms.

## Discussion

This case highlights the importance of combining clinical suspicion and laboratory and radiological investigations to diagnose and guide management of acquired hemophilia. It represents a rare condition with unique demographic and clinical findings, such as having female gender in the absence of personal or family history of bleeding disorders. In addition, unlike many published cases of acquired hemophilia, which present with mucocutaneous bleeding (e.g., epistaxis, hematuria, menorrhagia), this patient primarily had a spontaneous intramuscular hematoma in the biceps muscle.

The pathophysiology of acquired FVIII deficiency relies on developing IgG autoantibodies (mostly IgG1 and IgG4) that do not activate complement [6]. Measuring FVIII activity offers little to no benefit in managing hemorrhage in affected patients as they do not result in complete FVIII inactivation [7]. Differences between alloantibodies in congenital haemophilia and autoantibodies in acquired hemophilia are that the alloantibodies lead to complete elimination of factor VIII activity, while the acquired antibodies lead to partial elimination [6,8]. Laboratory investigations show an isolated prolonged aPTT. Hence, if prothrombin time (PT) is prolonged, other causes must be ruled out, reducing FVIII activity (FVIII:C) (<1% in 50% of cases; <5% in 75% of cases; <40% in 100% of cases). The Bethesda assay or enzyme-linked immunosorbent assay (ELISA) is used to detect antibodies [9].

In patients with active bleeding, several treatment options are available, regardless of inhibitor titer or residual FVIII activity. First-line therapies include bypassing agents such as recombinant activated FVII (rFVIIa; NovoSeven), activated prothrombin complex concentrate (aPCC; FEIBA), and recombinant porcine FVIII (rpFVIII). These agents are considered equally effective, with no demonstrated superiority among them. However, rpFVIII is typically avoided in patients with anti-rpFVIII inhibitor titers greater than 20 BU. Human FVIII concentrates are generally less effective than the aforementioned first-line therapies and are typically reserved for cases where those treatments are either unavailable or have failed-provided that the inhibitor titer is low. The use of desmopressin is not recommended due to its association with adverse effects such as hyponatremia [6].

Immunosuppressive therapy (IST) is used to reduce bleeding and accelerate the achievement of remission, as spontaneous remission is rare without it. Glucocorticoids are often used as monotherapy; however, in patients with a high inhibitor titer (greater than 20 BU), glucocorticoids are typically combined with either cyclophosphamide or rituximab [10].

Many reported cases of acquired hemophilia were associated with autoimmune conditions, recent pregnancy, or medication use [6]. However, this case does not possess any of the mentioned characteristics. The prolonged period between the onset of bleeding and giving birth, not taking any anticoagulant medications, and not having any positive autoimmune antibody testing, excludes the frequent etiological factors associated with acquired coagulation abnormalities, labeling it as an idiopathic acquired hemophilia, which makes up about 50% of the causes of acquired hemophilia [7].

According to a recent series, relapse was observed in 20% of 90 patients, with a median time of 7.5 months after remission. The highest relapse rate was seen in patients who received glucocorticoids alone, while the lowest was observed in those treated with a combination of glucocorticoids and rituximab. A higher relapse rate of 29% was noted in postpartum patients [11].

This case adds to the body of existing literature a deeper understanding of the nature and pathophysiology of acquired hemophilia. Through clinical and laboratory findings, this case reinforces the idea of an inhibitor (autoimmune factor) behind many cases of acquired hemophilia. Despite having a low inhibitor titer (3.1 BU), this patient had severe bleeding, which dropped hemoglobin levels, indicating no obvious relationship between the severity of symptoms and a high titer level [8]. The dramatic, rapid improvement (manifested by reducing aPTT and increasing the active FVIII) within a month following the initiation of immunosuppressant medications highlights the importance of rapid management of cases with similar circumstances and the variability of the response to treatment [9].

The decision behind initiating dual immunosuppressants is supported by many studies in the literature [12]. Other studies suggested prolonged rituximab as an alternative treatment if the cyclophosphamide-prednisone method had failed [13]. Monotherapy with corticosteroids alone was also suggested after successfully treating a 50-year-old woman with idiopathic AHA [14]. Despite the information provided by this case, limitations must be addressed. First, being a report of a single case, the findings and outcomes should not be generalized to all patients. In addition, prolonged follow-up was not possible due to the patient's preference and logistical circumstances. Thus, late complications and recurrence could not be assessed properly.

In conclusion, in the background of scarce information regarding the etiology and management of AHA, our case provides a solid ground for future research to further explore idiopathic acquired hemophilia.

## Conclusions

Idiopathic acquired hemophilia is a rare but potentially life-threatening bleeding disorder that requires a high index of suspicion for timely diagnosis. This case underscores the importance of integrating clinical presentation with targeted laboratory investigations, particularly in patients without a personal or family history of bleeding disorders. The absence of common risk factors highlights the need to consider AHA even in otherwise healthy individuals. Prompt initiation of combined immunosuppressive therapy led to rapid remission and normalization of coagulation parameters, emphasizing that early, aggressive treatment can result in favorable outcomes.
